# Comparison of the efficacy of enfortumab vedotin between patients with metastatic urothelial carcinoma who were treated with avelumab or pembrolizumab: real-world data from a multi-institutional study in Japan

**DOI:** 10.1007/s00432-024-05717-2

**Published:** 2024-04-09

**Authors:** Yosuke Hirasawa, Takahiro Adachi, Takeshi Hashimoto, Wataru Fukuokaya, Yuhei Koike, Yuji Yata, Kazumasa Komura, Taizo Uchimoto, Takuya Tsujino, Kazuki Nishimura, Kiyoshi Takahara, Masanobu Saruta, Kazutoshi Fujita, Mamoru Hashimoto, Hirotsugu Uemura, Ryoichi Shiroki, Takashi Azuma, Takahiro Kimura, Yoshio Ohno

**Affiliations:** 1 Department of Urology, Tokyo Medical University, 6-7-1, Nishi-shinjuku, Shinjuku-ku, Tokyo, 160-0023 Japan; 2 Department of Urology, The Jikei University School of Medicine, Tokyo, Japan; 3 Department of Urology, Osaka Medical and Pharmaceutical University, Osaka, Japan; 4 Department of Urology, Fujita-Health University School of Medicine, Aichi, Japan; 5 Department of Urology, Faculty of Medicine, Kindai University, Osaka, Japan

**Keywords:** Enfortumab vedotin, Pembrolizumab, Avelumab, Urothelial carcinoma, Bladder cancer

## Abstract

**Objectives:**

Enfortumab vedotin (EV) is a novel antibody–drug conjugate approved for metastatic urothelial carcinoma (UC) refractory to prior treatment with immune checkpoint inhibitors (ICIs). However, the difference in efficacy of EV after each ICIs and prognostic factors are not well known. We aimed to compare the efficacy of EV in patients with metastatic UC who were treated with avelumab or pembrolizumab and to identify the prognostic factors.

**Methods:**

The records of 100 patients with advanced metastatic UC who received EV after the administration of either avelumab or pembrolizumab were retrospectively collected from five academic hospitals in Japan.

**Results:**

The median follow-up period was 6.7 months. The median overall survival (OS) and progression-free survival (PFS) in the EV after avelumab/pembrolizumab group were not reached/14.7 months (p = 0.17) and 10.4/5.2 months (p = 0.039), respectively. The objective response rates (ORR) were 66.6% and 46.8% in EV after avelumab and EV after pembrolizumab groups, respectively (p = 0.14). Multivariate analysis identified histological variants, liver metastasis, low serum albumin levels, and high serum CRP level as significant poor prognostic factors. The median OS and PFS of cachexia patients with both low serum albumin levels and high serum CRP levels were 6.0 months and 0.93 months, respectively.

**Conclusion:**

PFS was superior in patients treated with EV after avelumab to EV after pembrolizumab. However, OS showed no significant difference between the two groups. Because the prognosis of patients with cachexia is extremely poor, the initiation of EV should be discussed in these patients.

## Introduction

According to the latest global cancer statistics, bladder cancer was the 10th most common cancer worldwide in 2020 (Estimated Number of New Cases 2020). Pure urothelial carcinoma (UC) is the most common histological form of bladder cancer, accounting for approximately 75% of all bladder cancer cases (Rogers et al. 2006). Non-UC tumors, such as squamous, neuroendocrine, micropapillary, and sarcomatoid cancers, are rare and mostly aggressive (Ismaili 2011). UC has a poor prognosis, and metastatic UC (mUC), which occurs in 5% of patients with UC, shows an extremely poor prognosis, with a reported 5-year survival rate of 4.6% (Saginala et al. 2020). Systemic therapy for mUC has undergone a significant transformation in recent years. First-line chemotherapy with methotrexate, vinblastine, adriamycin, and cisplatin (M-VAC) has been established as the standard of systemic therapy for mUC since 1988 (Nishio et al. 1988). In 2000, gemcitabine and cisplatin (GC) therapy was introduced and has demonstrated overall survival (OS) and objective response rates (ORRs) comparable to M-VAC therapy (Maase et al. 2000). GC therapy is also better tolerated with fewer severe adverse events, such as febrile neutropenia, making it more favored and widely used as the first-line systemic therapy for mUC (Maase et al. 2000). For a long time, vinflunine was the only approved second-line therapeutic agent for mUC with tumor progression (Oing et al. 2016); however, it showed only a 2.6-month improvement in OS in comparison with best supportive care (BSC) (p = 0.04) (Bellmunt et al. 2013, 2009). Other second-line chemotherapeutic agents, including docetaxel, taxanes, or cyclophosphamide, are yet to be established. By 2016, the treatment landscape for mUC transitioned to the next stage with the advent of immune checkpoint inhibitors (ICIs). Atezolizumab was approved by the U.S. FDA as the first PD-L1 inhibitor for second-line treatment in patients with mUC following chemotherapy with regimens such as GC or M-VAC. Subsequently, the PD-1 antibody pembrolizumab was approved by the U.S. FDA after showing significant OS benefit in a phase III randomized control trial (RCT) (Bellmunt et al. 2017). At present, five FDA-approved ICIs, namely, pembrolizumab, atezolizumab, duralumab, nivolumab, and avelumab, are available for second-line therapy after platinum-based chemotherapy for mUC. However, ICIs have some limitations; only approximately one-quarter to one-fifth of the patients respond to ICIs. Moreover, the ORR was approximately 20% in the second-line setting after progression under platinum-based chemotherapy (Bellmunt et al. 2017).

Enfortumab vedotin (EV) is an ADC conjugated to monomethyl auristatin E, a microtubule-disrupting agent that targets nectin-4, a cell adhesion molecule highly expressed in several solid tumors, including breast, lung, gastric, and UC (Bouleftour et al. 2022). The confirmatory phase III trial EV-301 demonstrated a median OS of 12.88 months, with a median progression-free survival (PFS) period of 5.55 months versus 3.71 months after prior chemotherapy and ICI therapy (Powles et al. 2021). Patients who respond with stable disease (SD) or show improvement after chemotherapy transition to avelumab treatment, while those who experience disease progression (PD) switch to pembrolizumab treatment. Furthermore, if PD occurs after these treatments, transition to EV treatment is considered. However, few trials have compared the efficacy of EV in these treatment arms. The superior efficacy of EV in patients who were previously treated with either avelumab or pembrolizumab has not been sufficiently verified. Therefore, this study aimed to compare the efficacy of EV in patients with mUC who had previously received pembrolizumab or avelumab.

## Materials and methods

This multi-institutional study was approved by the Institutional Review Board and complied with the 1964 Declaration of Helsinki and its later amendments. We identified 109 consecutive patients with advanced UC who received EV at five institutions (Jikei University School of Medicine, Osaka Medical and Pharmaceutical University, Fujita Health University School of Medicine, Kindai University Faculty of Medicine, and Tokyo Medical University) between December 2022 and June 2023. Patients who did not receive any ICIs before the initiation of EV therapy (n = 3) and those who did not undergo radiographic examinations after the initiation of EV therapy (n = 6) were excluded. Thus, 100 patients were enrolled in this study. All patients were histopathologically diagnosed with UC and radiologically confirmed to have PD after receiving ICIs as second-line therapy. Radiological evaluations were generally performed using computed tomography before and after every three–six cycles of EV; however, evaluations were also performed as needed when clinicians thought it necessary, for example, when the clinical symptoms worsened. Radiological analysis was based on the Response Evaluation Criteria in Solid Tumors (RECIST), version 1.1 (Eisenhauer et al. 2009). For each metastatic lesion, the best response was classified as follows: (1) complete response (CR; disappearance or reduction to a short-axis diameter < 10 mm for all LN metastases), (2) partial response (PR; > 30% reduction), (3) SD (neither CR, PR, nor PD), and (4) PD (> 20% growth). Laboratory data were collected within 1 month of the initial administration of EV therapy. EV was administered at a dose of 1.25 mg per kg of body weight as a single intravenous infusion over 30 min on days 1, 8, and 15 of a 4-week cycle.

We defined the “EV after avelumab group” as patients who received avelumab followed by EV, and the “EV after pembrolizumab group” as those who received pembrolizumab followed by EV. The primary endpoint was OS, and the secondary endpoints were PFS, ORR, and disease control rate (DCR).

The significance tests used to compare the two groups were the Student’s t test for continuous variables and the χ^2^ test for categorical variables. Kaplan–Meier analysis and the log-rank test were used to obtain and compare the OS and PFS between the two groups, respectively. Multivariate analysis was performed to identify independent prognostic variables affecting the OS of all patients by using a stepwise Cox proportional-hazards regression model. Receiver operating characteristic curves and the Youden index were used to determine the optimum cut-off values of the continuous variables. All statistical analyses were performed using R 3.1.0 for Windows (R Foundation for Statistical Computing, Vienna, Austria; http://www.r-project.org/). All parameters in this study with a p < 0.05 were considered statistically significant.

## Results

**Fig. 1:**
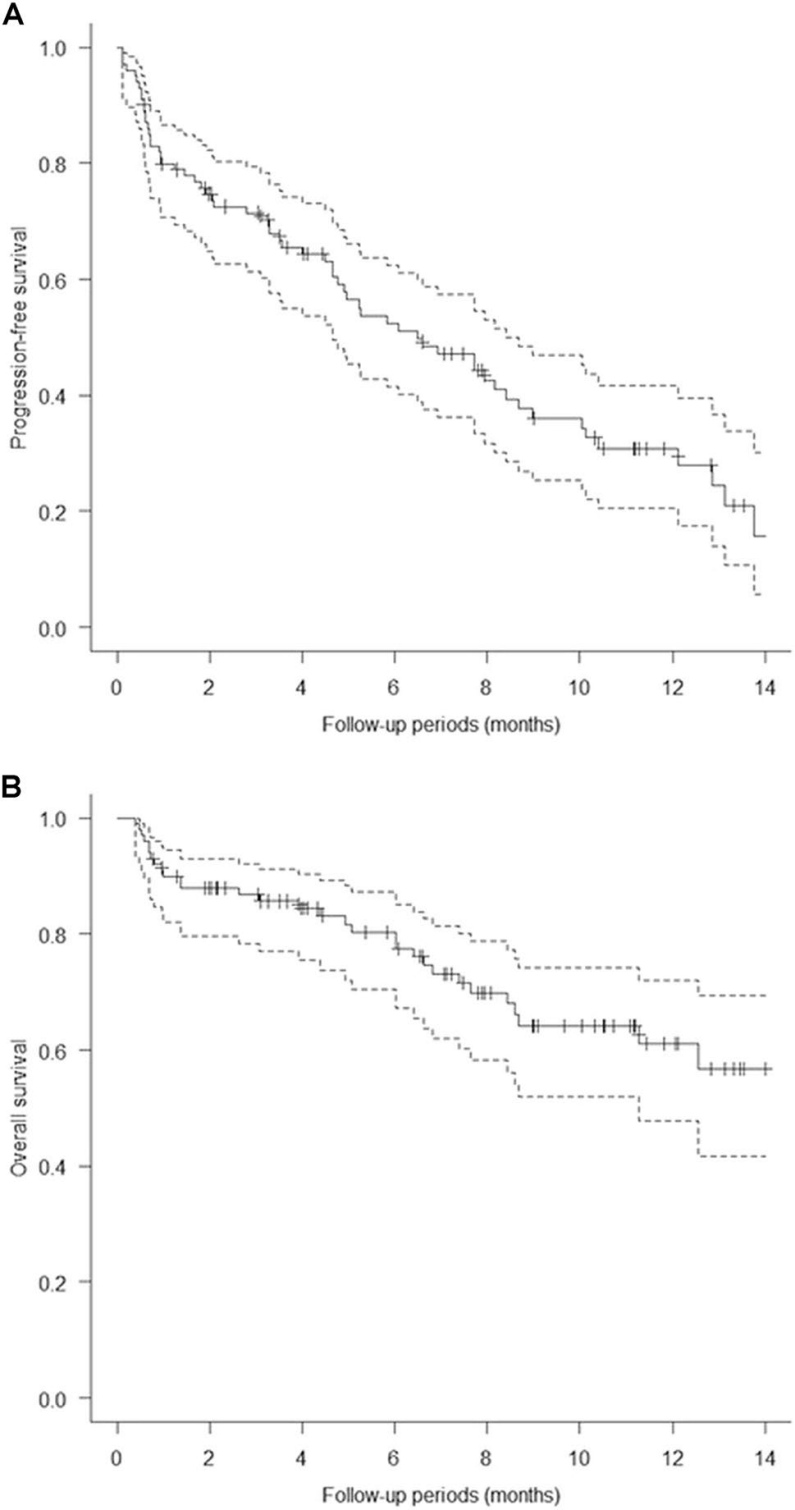
**A** Kaplan–Meier curves of progression-free survival of all patients (n = 100). **B** Kaplan–Meier curves of overall survival of all patients (n = 100)

**Fig. 2:**
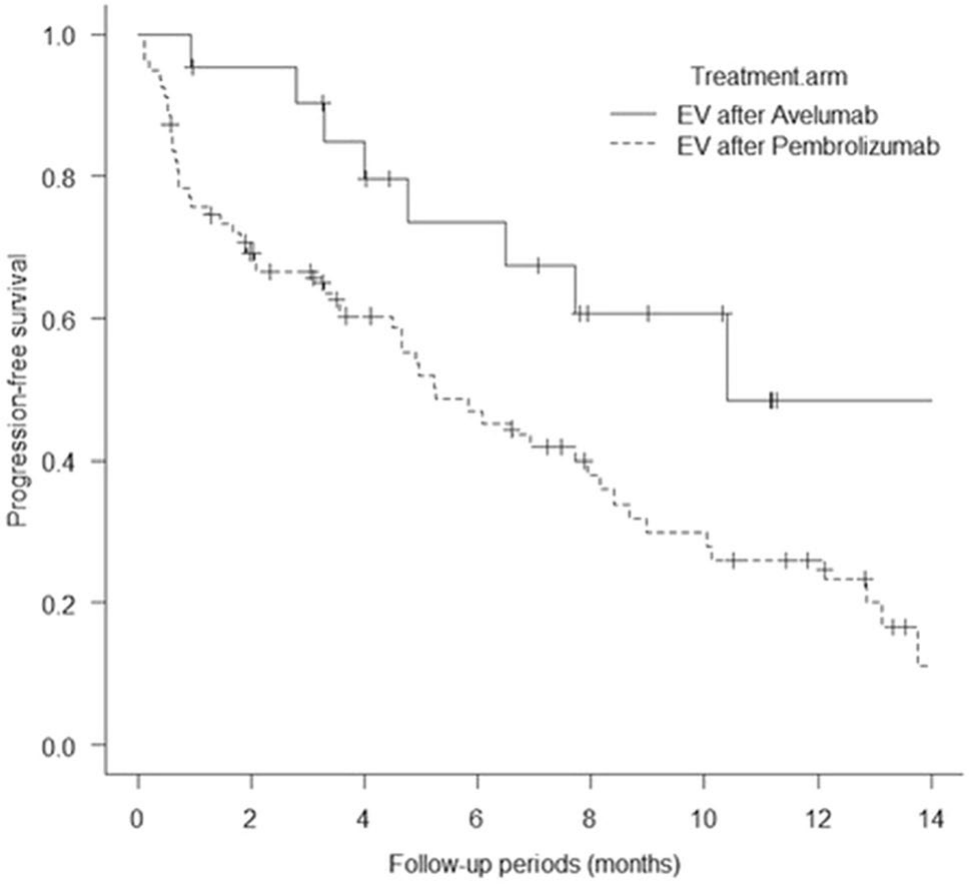
Kaplan–Meier curves of progression-free survival according to treatment group. (log-rank test, p = 0.0392)

**Fig. 3:**
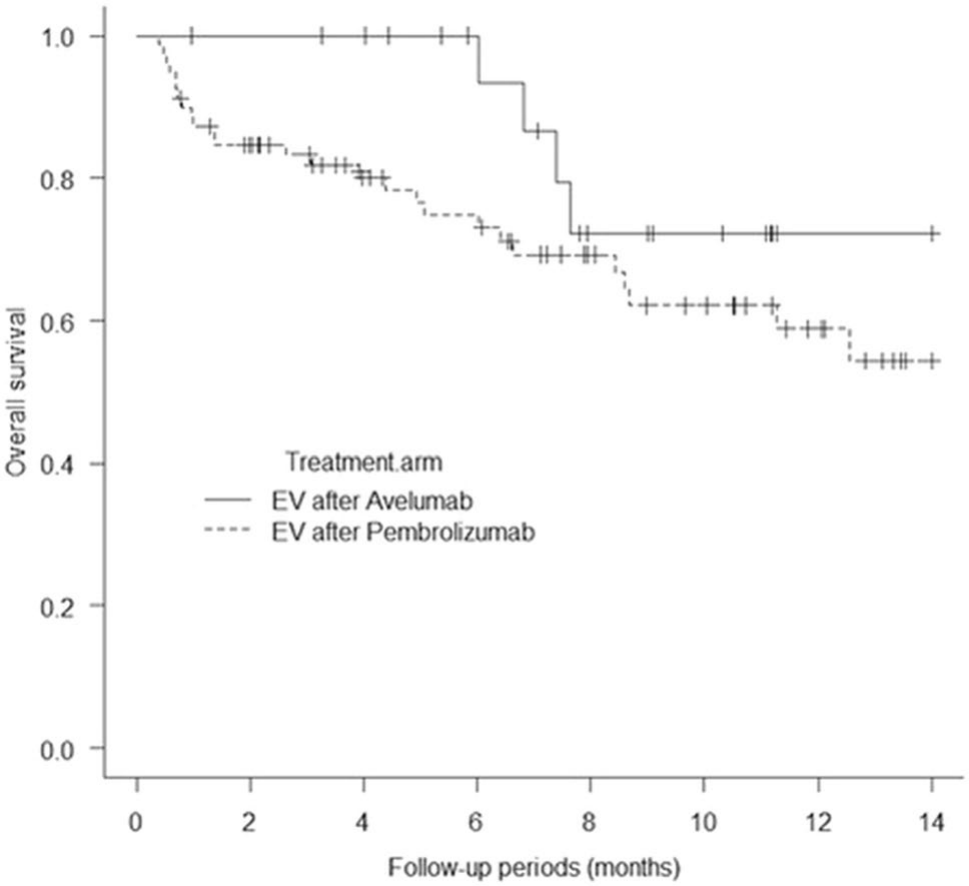
Kaplan–Meier curves of overall survival according to treatment group. (log-rank test, p = 0.176)

**Fig. 4:**
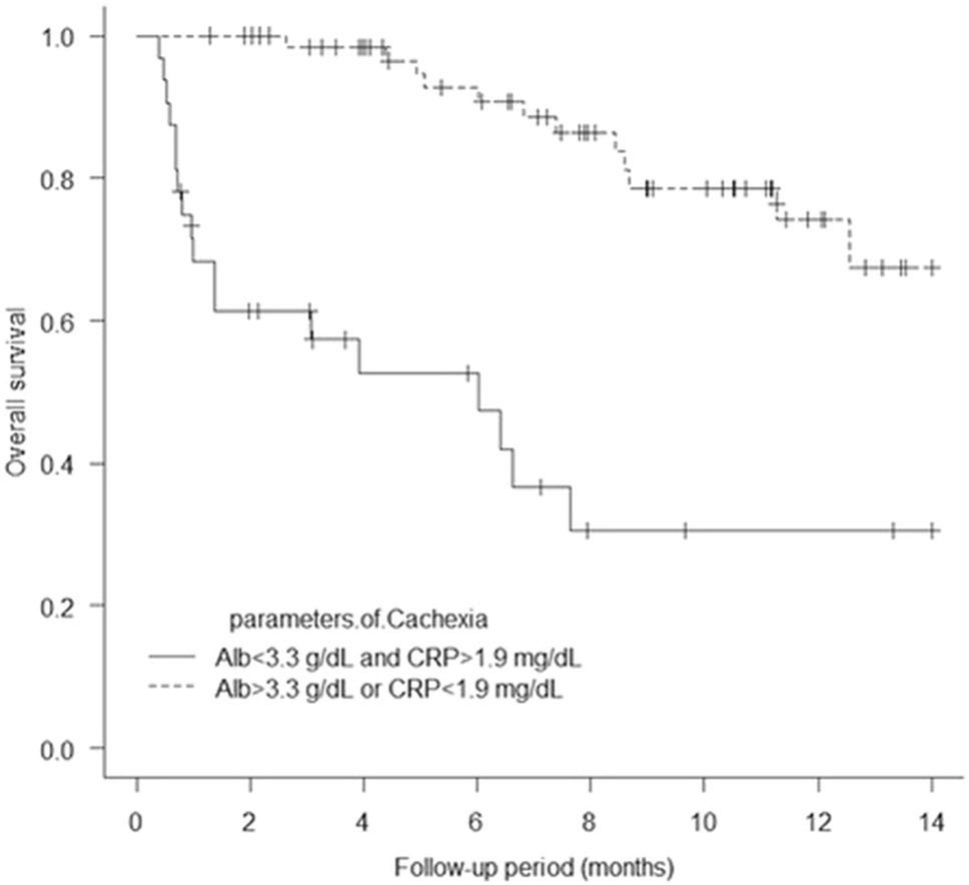
Kaplan–Meier curves of overall survival in patients with and without cachexia who were treated with enfortumab vedotin after ICIs. (log-rank test, p = 0.000000011)

**Fig. 5:**
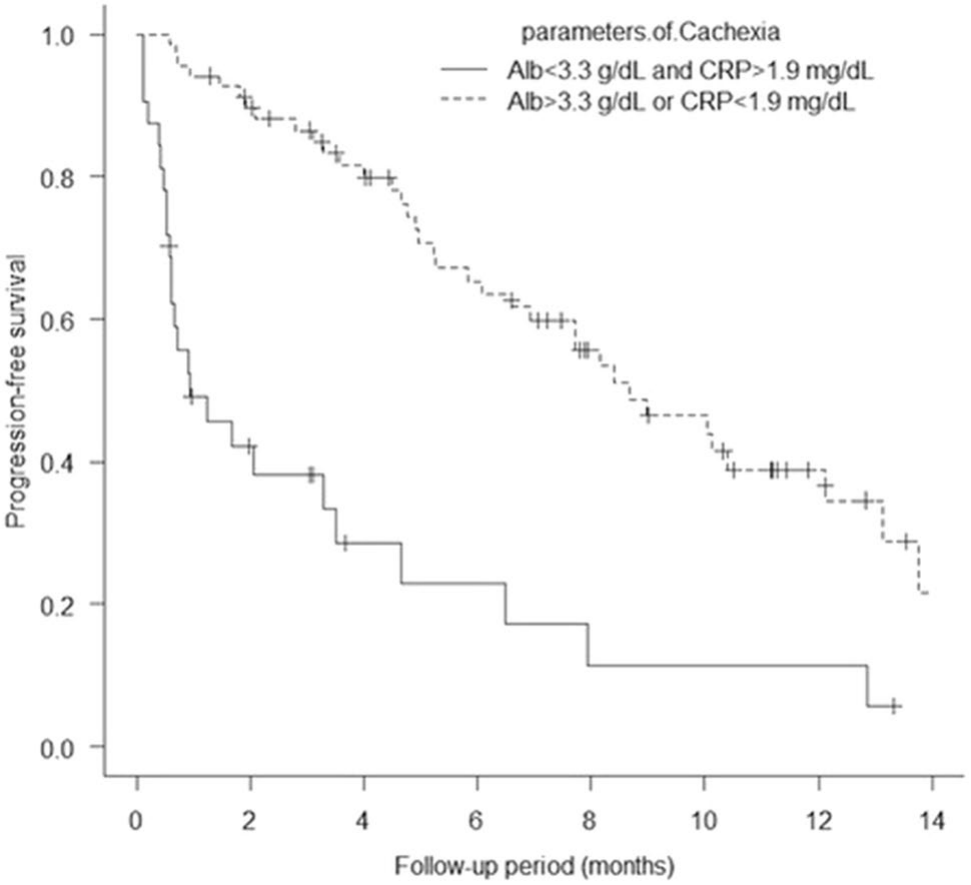
Kaplan–Meier curves of progression-free survival in patients with and without cachexia who were treated with enfortumab vedotin after ICIs. (log-rank test, p = 0.000000070)

One hundred patients from five academic institutions in Japan who had been treated with EV after avelumab or pembrolizumab were enrolled in the present study. Baseline patient characteristics are shown in Table 1. The mean patient age was 73.6 years, and most (67%) of the patients were male; 54% of the patients had a history of smoking or were currently smoking. Visceral metastasis was observed in 52.3% of the patients in the EV after avelumab group and 67% of those in the EV after pembrolizumab group (p = 0.30). Liver metastasis was present in 19% and 16.4% of the patients in the two groups (p = 0.75), respectively. The two groups showed no significant differences in patient characteristics, except for sex (Table 1). The median follow-up period was 7.6 months (range: 0.97–15.5 months) in the EV after avelumab group and 6.4 months (range: 0.4–16.6 months) in the EV after pembrolizumab group. A total of 31 deaths (31%) occurred, including four in the EV after avelumab group (19%) and 27 in the EV after pembrolizumab group (34.1%). The median PFS of all 100 patients was 6.5 months (Fig. 1A). The median PFS was 10.4 months (95% CI: 0.19–0.72) in the EV after avelumab group and 5.2 months (95% CI: 0.36–0.59) in the EV after pembrolizumab group, and was significantly better in the EV after avelumab group (p = 0.039; Fig. 2). The median OS of all 100 patients was 14.7 months. (Fig. 1B). The median OS was not determinable in the EV after avelumab group, but was 14.7 months (95% CI: 0.21–0.63) in the EV after pembrolizumab group. Nonetheless, it did not differ significantly between the two groups (p = 0.17; Fig. 3). CR/PR was achieved in 14.2%/52.3% of the patients in the EV after avelumab group and 2.5%/44.3% of those in the EV after pembrolizumab group (Table 2). The ORR was 66.6% in the EV after avelumab group and 46.8% in the EV after pembrolizumab group (p = 0.14) (Table 2). The DCR was 85.7% in the EV after avelumab group and 70.8% in the EV after pembrolizumab group (p = 0.26) (Table 2). Multivariate analysis using the Cox proportional-hazards model identified histological variant (HR: 21.4, p = 0.000017), liver metastasis (HR: 4.5, p = 0.0018), low serum albumin (Alb) level (Alb < 3.3 g/dL; (HR: 4.2, p = 0.0031), and high serum C-reactive protein (CRP) level (CRP > 1.9 mg/dL; HR: 3.3, p = 0.011) as significant independent poor prognostic factors in the EV after ICI groups (Table 3). Thirty-two patients (32%) with cachexia met the criteria of both low serum Alb levels (Alb < 3.3 g/dL) and high serum CRP levels (CRP > 1.9 mg/dL) in all patients. The median OS and PFS of these patients were 6.0 months (95% CI, 0.27–0.64) (Fig. 4) and 0.93 months (95% CI, 0.30–0.65) (Fig. 5), respectively.

**Table 1:** Baseline patient characteristics

	All	Prior Anti-PD-(L)1 therapy; Avelumab group	Prior Anti-PD-(L)1 therapy; Pembrolizumab group	p-value
Number of patients	100	21	79	
Age, years (Mean, SD)	73.6 ± 8.6	74.3 ± 6.7	73.5 ± 9.1	0.69
Age ≥ 75 years, no. (%)	49 (49%)	10 (47.6%)	39 (49.3%)	1.0
Male sex	67 (67%)	9 (42.8%)	58 (73.4%)	0.016
ECOG				0.34
0–1	82 (82%)	19 (90.4%)	63 (79.7%)	
≥ 2	18 (18%)	2 (9.5%)	16 (20.2%)	
Tobacco use, no. (%)				0.23
Former user	50 (50%)	8 (38%)	42 (53.1%)	
Current user	4 (4%)	0	4 (5%)	
Never user	46 (46%)	13 (61.9%)	33 (41.7%)	
Primary tumor site				0.62
Bladder	57 (57%)	11 (52.3%)	46 (58.2%)	
Upper urinary tract	42 (42%)	10 (47.6%)	32 (40.5%)	
Missing	1 (1%)	0	1 (1.2%)	
Histologic type				0.83
Urothelial carcinoma	78 (78%)	17 (76.1%)	61 (77.2%)	
Urothelial carcinoma with variant histology	5 (5%)	0	5 (6.3%)	
Other	9 (9%)	2 (9.5%)	7 (8.8%)	
Missing	8 (8%)	2 (9.5%)	6 (7.5%)	
Symptomatic disease	60 (60%)	11 (52.3%)	49 (62%)	0.61
Missing	1 (1%)	1 (4.7%)	0	
Prior surgery or radiation				0.52
Surgery	58 (58%)	10 (47.6%)	48 (60.7%)	
Radiation	8 (8%)	2 (9.5%)	6 (7.5%)	
Surgery + Radiation	3 (3%)	0	3 (3.7%)	
None	31 (31%)	9 (42.8%)	22 (27.8%)	
Previous chemotherapy				0.26
Cisplatin	67 (67%)	17 (80.9%)	50 (63.2%)	
Carboplatin	23 (23%)	4 (19%)	19 (24%)	
Other	8 (8%)	0	8 (10.1%)	
NA	2 (2%)	0	2 (2.5%)	
Metastatic disease				
Visceral metastasis	64 (64%)	11 (52.3%)	53 (67%)	0.30
Liver	17 (17%)	4 (19%)	13 (16.4%)	0.75
Lung	49 (49%)	6 (28.5%)	43 (54.4%)	0.049
Bone	28 (28%)	6 (28.5%)	22 (27.8%)	1.0
Lymph node	65 (65%)	13 (61.9%)	52 (65.8%)	0.79
Number of metastasis sites (mean, standard deviation)	1.9 ± 1.0	1.7 ± 1.3	2.0 ± 0.91	0.31
Number of visceral metastasis sites (mean, standard deviation)	0.8 ± 0.68	0.61 ± 0.66	0.84 ± 0.68	0.17
Perioperative therapy				0.84
Neoadjuvant chemotherapy	6 (6%)	1 (4.7%)	5 (6.3%)	
Adjuvant chemotherapy	6 (6%)	2 (9.5%)	4 (5%)	
Salvage chemotherapy	86 (86%)	18 (85.7%)	68 (86%)	
Best response among patients who previously received chemotherapy				0.0035
CR	6 (6%)	1 (4.7%)	5 (6.3%)	
PR	26 (26%)	10 (47.6%)	16 20.2%)	
SD	30 (30%)	9 (42.8%)	21 (26.5%)	
PD	31 (31%)	1 (4.7%)	30 (37.9%)	
NA	7 (7%)	0	7 (8.8%)	
Best response among patients				0.97
who previously received ICIs				
CR	4 (4%)	0	4 (5%)	
PR	15 (15%)	3 (14.2%)	12 (15.1%)	
SD	19 (19%)	4 (19%)	15 (18.9%)	
PD	60 (60%)	13 (61.9%)	47 (59.4%)	
Follow-up period, months (Median, range)	6.7 (0.4–16.6)	7.6 (0.97–15.5)	6.4 (0.4–16.6)	0.35

*PD-(L)1* programmed death-1 receptor/programmed death ligand-1; *ECOG* Eastern Cooperative Oncology Group; *ICIs* immune checkpoint inhibitors; *CR* complete response; *PR* partial response; *SD* stable disease; *PD* progressive disease; *NA* not applicable

**Table 2:** Objective response rate

Variable	All	EV after avelumab group	EV after pembrolizumab group	p-value
No. of patients	100	21	79	
Best response assessed using RECIST criteria				0.11
CR	5 (5%)	3 (14.2%)	2 (2.5%)	
PR	46 (46%)	11 (52.3%)	35 (44.3%)	
SD	23 (23%)	4 (19%)	19 (24%)	
PD	26 (26%)	3 (14.2%)	23 (29.1%)	
ORR	51 (51%)	14 (66.6%)	37 (46.8%)	0.14
DCR	74 (74%)	18 (85.7%)	56 (70.8%)	0.26

DCR = CR + PR + SD

ORR = CR + PR

*EV* enfortumab vedotin; *CR* complete response; *PR* partial response; *SD*, table disease; *PD* progressive disease; *ORR* objective response rate; *DCR* disease control rate

**Table 3:** Univariate and multivariate Cox regression models for overall survival in patients with metastatic urothelial carcinoma who were treated with enfortumab vedotin after ICI therapy

Variable	Univariate analysis Hazard ratio (95% confidence interval)	p-value	Multivariate analysis Hazard ratio (95% confidence interval)	p-value
Male sex (vs. female)	0.95 (0.44–2.0)	0.90		
ECOG > 2 (vs. < 0–1)	2.6 (1.2–5.8)	0.013		
Smoking (vs. never)				
Former	1.4 (0.67–2.9)	0.36		
Current	0.99 (0.12–7.6)	0.99		
Symptom (vs. none)	2.3 (1.0–5.5)	0.044		
Pembrolizumab as prior treatment (vs Avelumab)	2.0 (0.71–5.8)	0.18		
Histological type (vs. urothelial carcinoma)				
Other	3.8 (1.5–9.6)	0.0048	5.4 (1.9–14.9)	0.00093
UC with variant	6.7 (2.1–21.1)	0.00097	21.4 (5.2–86.6)	0.000017
Missing	0.50 (0.067–3.8)	0.51	0.32 (0.04–2.6)	0.29
BOR of chemotherapy PR-CR (vs. PD-SD)	0.40 (0.16–0.98)	0.046		
BOR of immunotherapy PR-CR (vs. PD-SD)	0.37 (0.11–1.2)	0.10		
Number of visceral metastatic site (vs 0)				
1	0.81 (0.35–1.8)	0.62		
2	2.5 (1.0–6.4)	0.045		
Liver metastasis	2.1 (0.95–4.8)	0.064	4.5 (1.7–11.9)	0.0018
Alb < 3.3 g/dL (vs. > 3.3 g/dL)	3.6 (1.7–7.6)	0.00058	4.2 (1.6–10.9)	0.0031
CRP > 1.9 mg/dL (vs. < 1.9 mg/dL)	6.0 (2.6–13.5)	0.000015	3.3 (1.3–8.3)	0.011
Hb < 11 g/dL (vs. > 11 g/dL)	2.1 (1.0–4.4)	0.049		
NLR > 3.8 (vs. < 3.8)	3.8 (1.5–9.4)	0.0028		

*ICIs* Immune checkpoint inhibitors; *ECOG* Eastern Cooperative Oncology Group; *UC* Urothelial carcinoma; *CR* complete response; *PR* partial response; *SD* stable disease; *PD* progressive disease; *Alb* albumin level; *CRP* C-reactive protein level; *NLR* neutrophil-to-lymphocyte ratio

## Discussion

To the best of our knowledge, this is the first multi-institutional retrospective study to compare the efficacy of EV between patients with mUC who had been previously treated with avelumab or pembrolizumab.

Herein, we report the data of 100 patients from five academic hospitals in Japan. The median OS in our study was 14.7 months (Fig. 1B), which is comparable to the OS in the UNITE study (14.4 months) (Koshkin et al. 2022), and slightly longer than those reported in the EV-201 (11.7 months) (Rosenberg et al. 2019) and EV-301 (12.8 months) (Bouleftour et al. 2022) studies. When analyzed by each arm, the median OS in the EV after avelumab group was not applicable, and that in the EV after pembrolizumab group was 14.7 months. The 12-month survival rates in the EV after avelumab and pembrolizumab groups were 72.2% and 58.8%, respectively. The two groups showed no significant difference in OS (p = 0.17) (Fig. 3). Furthermore, based on the multivariate analysis, ICI therapy prior to EV was not a significant factor affecting OS (Table 3). In the UNITE study, Koshkin et al. reported that the expression of PD-L1 was not significant in affecting OS in patients with advanced UC who were treated using EV (Koshkin et al. 2022). The current study is the first to report that the difference in using PD-1 and PD-L1 inhibitors in prior treatment does not affect OS in patients with advanced UC who were treated using EV. Our results showed a slightly better OS than those of the EV-201 and EV-301 studies. However, our patient population had a lower percentage of individuals with visceral (64%) and liver (17%) metastases than the EV-301 group (77.7% and 30.9%, respectively). Conversely, our study included a higher percentage of patients with lymph node metastasis (22%) than the EV-301 group (11.3%). Thus, our study may have included a relatively larger population of patients with a better prognosis. In terms of PFS, the median PFS in our study was 6.5 months (Fig. 1A), which is comparable to the PFS in the UNITE study (6.8 months) (Koshkin et al. 2022), and was slightly longer than those in the EV-201 study (5.8 months) (Rosenberg et al. 2019) and the EV-301 study (5.55 months) (Bouleftour et al. 2022). When analyzed by each arm, the median PFS in the EV after avelumab group was 10.4 months and that in the EV after pembrolizumab group was 5.2 months. The PFS in the EV after avelumab group was significantly superior to that in the EV after pembrolizumab group (p = 0.039) (Fig. 2). These findings might be attributed to the fact the avelumab arm included patients who responded to first-line chemotherapy (SD or better), whereas the pembrolizumab arm included patients who experienced disease progression (PD). Thus, the two groups may have shown differences in tumor progression rate, tumor burden, and tumor quantity at the initiation of EV treatment. In the present study, the actual number of metastases, number of metastases, and visceral metastases tended to be lower in the EV after avelumab group than in the EV after pembrolizumab group, although no statistically significant differences were observed (Table 1). Moreover, the number of patients with lung metastases in addition to liver or bone metastases was higher in the EV after pembrolizumab group (Table 1). However, despite the superior PFS in the avelumab arm, the two groups showed no significant difference in OS. Notably, approximately 6 months after initiating EV treatment, the two groups showed no substantial difference in OS in the present study (Fig. 3). The pembrolizumab group included more patients with advanced tumors, resulting in a subgroup of patients who quickly succumbed to cancer in the early stages, leading to a poorer OS trend. However, the avelumab group also showed gradual worsening of disease progression, which may ultimately result in a less favorable long-term OS outcome. More recently, Nizam et al. reported at the 2024 American Society of Clinical Oncology Genitourinary (ASCO GU) meeting that patients with advanced UC treated with EV following maintenance therapy using Avelumab had outcomes consistent with data for EV in chemotherapy- and ICI-refractory advanced UC. (Nizam et al. 2024) Both their study and ours support the use of EV as third-line therapy after progression on maintenance therapy using Avelumab. However, validation in larger cohorts is required.

Multivariate analysis indicated that histological variants, liver metastasis, low serum Alb levels (Alb < 3.3 g/dL), and high serum CRP levels (CRP > 1.9 mg/dL) were significant independent poor prognostic factors (Table 3). All of these are known poor prognostic factors for UC; however, we focused on low Alb and high CRP levels. Low Alb (Cong et al. 2022) (Liu et al. 2021) (Liu et al. 2022) and high CRP levels (Cong et al. 2022) (Fearon et al. 2006) (McMillan 2008) (Marsik et al. 2008) (Hilmy et al. 2005) are typical indicators of cachexia. In the present study, 32 patients (32%) met the criteria of low Alb (Alb < 3.3 g/dL) and high CRP (CRP > 1.9 mg/dL) levels, and approximately half of them experienced cancer-related death within 3 months of starting treatment, with 70% succumbing to cancer within 7 months (Fig. 4). The median OS and PFS of the patients with cachexia were 6.0 months (95% CI, 0.27–0.64; Fig. 4) and 0.93 months (95% CI, 0.30–0.65; Fig. 5), respectively. This can be explained by the fact that the proportion of patients with cachexia due to advanced UC is higher among patients who are starting EV as a third-line treatment. As the prognosis of patients with cachexia is extremely poor, the initiation of EV treatment in these patients should be well discussed, especially within the context of payer restrictions and limited access in emerging markets. In addition, although the Bellmunt Risk Score has been reported as a prognostic factor in patients with UC who were treated with chemotherapy or immunotherapy (Bellmunt et al. 2010) (Abuhelwa et al. 2022), in our multivariate analysis, Hb and ECOG PS were not significant prognostic factors (Table 3). Thus, there is a need to develop new risk models to predict prognosis at the time of administering the EV.

The current study is limited by its retrospective nature and variable follow-up protocols resulting from its multi-institution design. In addition, it lacked data regarding the tumor burden in the two groups. Further research is needed to investigate the intergroup differences in the tumor burden and patient backgrounds. Moreover, while we intended to analyze the prognostic factors for each group, we could not analyze the prognostic factors of patients treated with avelumab followed by EV because of the small sample size in that group. Large-scale and long-term studies are needed to clarify these points.

## Conclusions

The efficacy of EV in patients with mUC in this study was comparable to that of previous studies. The PFS was superior in patients treated with EV after avelumab to those treated with EV after pembrolizumab. However, OS showed no significant difference between the two groups. Because the prognosis of patients with cachexia is extremely poor, the initiation of EV should be well discussed in these patients.

## Data Availability

The data from this study can be accessed by academic and commercial partners upon a reasonable request, subject to Institutional Review Board (IRB) approval and a data use agreement. For further details or to reanalyze the study's data, contact the Lead Contact at kazumasa.komura@ompu.ac.jp.
